# Neural mechanisms of food preference and reward processing: a review of multifaceted influencing factors and intervention strategies

**DOI:** 10.3389/fnut.2026.1823431

**Published:** 2026-04-10

**Authors:** Xiaomeng Gao, Yihan Wu, Ronglian Zheng, Yining Kou, Huili Xing, Kun Li, Meng Zhang

**Affiliations:** 1 Faculty of Psychology, Tianjin Normal University, Tianjin, China; 2 Department of Psychology, Henan Medical University, Xinxiang, Henan, China; 3 Henan Provincial People's Hospital, Zhengzhou, Henan, China; 4 Sichuan Academy of Medical Sciences – Sichuan Provincial People's Hospital, Chengdu, Sichuan, China; 5 College of Education, Shanghai Normal University, Shanghai, China; 6 Jing Hengyi School of Education, Hangzhou Normal University, Hangzhou, China; 7 Shandong Daizhuang Hospital, Jining, Shandong, China; 8 Jining Key Laboratory of Neuromodulation, Jining, Shandong, China; 9 Department of Psychology, University of Chinese Academy of Sciences, Beijing, China

**Keywords:** cognitive neuroscience, food preference, influencing factors, intervention strategies, reward processing

## Abstract

Food preference and reward processing are crucial determinants of dietary behavior and energy balance, and their dysregulation is closely linked to major public health problems such as obesity and eating disorders. This narrative review synthesizes current evidence on the cognitive and neural foundations of food preference and reward processing, examines multi-level determinants ranging from individual biology to broader social context, and discusses major intervention strategies. Research in cognitive neuroscience points to a distributed network involving the midbrain dopamine system, prefrontal cortex, amygdala, insula, and hypothalamus, which together regulate food “wanting” and “liking” as well as related processes of valuation, decision-making, and inhibitory control. These mechanisms are further shaped by genetic susceptibility, physiological state, developmental stage, stress, socioeconomic conditions, social learning, and cultural context. Building on this framework, current interventions target different components of the reward-control system, including appetite and gut-brain signaling (e.g., GLP-1-based approaches), cognitive control and behavioral regulation, and post-surgical changes in hormonal and neural responses. We also highlight emerging digital and real-world intervention models, including personalized and just-in-time approaches, while noting that their evidence base remains developing. Overall, this review emphasizes the need for integrative, mechanistically informed, and personalized strategies to improve dietary health.

## 1 Introduction

In recent decades, there has been a sharp increase in the global prevalence of obesity and related metabolic disorders, posing a significant public health challenge (1–5). The modern obesogenic environment is characterized by the widespread availability of high-energy, palatable foods, which greatly challenges the physiological systems evolved by humans to efficiently store energy (6–9). Individual dietary behaviors, particularly food preferences and responses to food reward, are central drivers of energy intake (10–13). Understanding the cognitive and neural mechanisms underlying these behaviors is crucial for explaining why many individuals struggle to maintain energy balance when faced with abundant food choices.

Food preference is not simply a matter of taste experience but a complex cognitive process involving learning, memory, emotion, and decision-making (14). It determines which food individuals choose among various options. Closely related to this is reward processing, a fundamental neurobiological process often divided into two core components: “liking” and “wanting” (15, 16). “Liking” refers to the hedonic pleasure experienced when consuming food (17–20), while “wanting” is the motivational drive generated upon encountering food or related cues (21, 22). Although these processes are interrelated, they are mediated by different neural circuits, with their imbalance considered a key factor leading to overeating and addictive behaviors (23–26).

The brain processes food rewards and guides food choices through a complex network that integrates internal physiological signals (such as hunger, satiety) and external environmental cues (27). This network is centered around the reward system but is also modulated by higher cognitive functions (such as executive function and inhibitory control) (28–31). When these cognitive control functions weaken or when the drive of the reward system is too strong, individuals may be more inclined to choose high-calorie foods for immediate gratification, disregarding their long-term health consequences (10).

However, food preferences and reward processing are not solely determined by intrinsic neurobiology. They are dynamically shaped by multiple levels of factors from individual to societal (32, 33). At the individual level, genetic background (34), age (35), stress levels (36), and health status (37) all influence the brain’s response to food. At the societal level, socioeconomic status (38), cultural norms (39), gender (40, 41), social interactions (42), and learning processes (43) play crucial roles. For instance, positive emotions can be transmitted between individuals through social interactions, even affecting food preferences (42).

Given this complexity, the present review has three aims. First, we synthesize current evidence on the cognitive and neural foundations of food preference and reward processing, with a focus on key brain regions, neurotransmitters, hormones, and cognitive processes. Second, we examine the multi-level factors that shape these mechanisms, spanning individual biology, developmental and psychological influences, and broader social and environmental contexts. Third, we discuss existing intervention strategies—including pharmacological, behavioral, surgical, and emerging digital approaches—in relation to the underlying reward-control framework. By integrating these perspectives, we aim to clarify the current state of knowledge, identify major unresolved questions, and outline priorities for future research and translational application.

This article is intended as a narrative review that integrates evidence across neurobiological, cognitive, behavioral, and socio-environmental domains. Our goal is conceptual synthesis rather than exhaustive study capture or quantitative aggregation. Accordingly, we focus on influential theoretical models, representative empirical findings, and translational implications that help explain how food preference and reward processing emerge from interactions between reward, homeostatic, and control systems across different levels of analysis.

## 2 Cognitive and neural foundations of food preferences and reward processing

**Figure 1:**
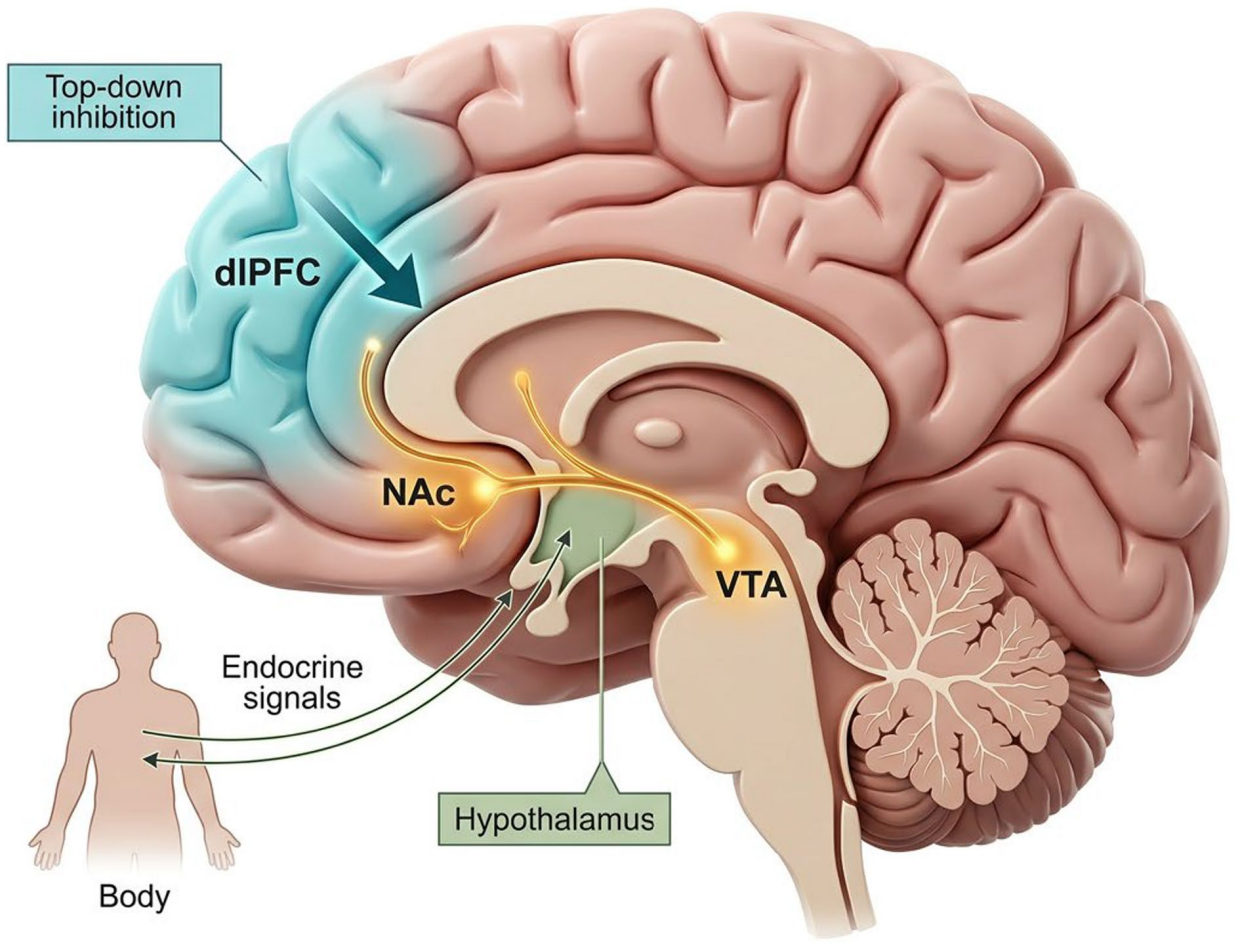
The neural architecture of food reward and executive control. The figure illustrates the dynamic interaction between bottom-up reward signaling and top-down regulatory control during food-related decision-making. Mesolimbic pathways centered on the VTA–NAc circuit contribute primarily to motivational salience and cue-triggered “wanting,” whereas prefrontal regions, particularly the dlPFC, support inhibitory control and goal-directed regulation. The OFC/vmPFC contribute to value representation, and the hypothalamus integrates homeostatic signals such as hunger and satiety. Conceptually, adaptive eating behavior depends on the coordinated balance among these systems, whereas maladaptive eating may emerge when reward drive persistently outweighs executive regulation.

The neural basis of food preference and reward processing is a complex network involving multiple brain regions and neurotransmitter systems. It integrates sensory, emotional, motivational, and cognitive information to ultimately guide behavioral decisions (Figure 1).

### 2.1 Core brain regions and neural circuits

The mesolimbic dopamine system, particularly the projection from the ventral tegmental area (VTA) to the nucleus accumbens (NAc), is considered central to reward processing and motivated behavior (44–47). Dopamine release primarily encodes reward prediction errors and the salience of motivation, driving individuals to seek and obtain rewards, closely linked to the concept of “wanting” (48–50). Dysfunction in the dopamine pathways in obese individuals may lead to reduced sensitivity to food rewards and compensatory overeating (51–53). The escalating availability of ultra-processed foods (UPFs) presents a significant challenge to the mesolimbic dopamine system. Chronic exposure to the synergistic combination of refined carbohydrates and fats in UPFs can trigger neuroplastic adaptations akin to substance use disorders, characterized by a progressive downregulation of striatal dopamine D2 receptors. This induction of a hypo-dopaminergic state leads to an upward shift in reward thresholds (54), driving compensatory overeating as individuals seek to overcome a diminished hedonic response to standard nutritional stimuli. The NAc, serving as an interface between cognition, emotion, and action, plays a crucial role in integrating information from different brain regions to optimize behavioral choices (55).

The prefrontal cortex (PFC) is central to reward regulation because it supports value computation, goal-directed choice, and inhibitory control (56–59). Within this system, the orbitofrontal cortex (OFC) and ventromedial prefrontal cortex (vmPFC) primarily encode the subjective value of food options by integrating sensory features, prior experience, and current physiological needs (60–63), whereas the dorsolateral prefrontal cortex (dlPFC) and ACC contribute more strongly to self-control, conflict monitoring, and the implementation of long-term goals (64–72). Thus, prefrontal regions do not merely evaluate food rewards; they also influence whether immediate reward signals are translated into action or restrained in favor of broader health goals.

The amygdala, traditionally associated with fear and negative emotions (73–77), is increasingly recognized for its crucial role in processing reward-related information (78–80), particularly in learning and memory of reward-associated cues (78, 79). It assigns emotional and motivational significance to stimuli (81–83), influencing attention (84, 85) and food choices (86, 87).

The hypothalamus, a traditional center for energy homeostasis, regulates hunger and satiety by integrating peripheral hormone signals such as leptin and insulin (88–91). However, hypothalamic neurons, especially orexin/hypocretin neurons in the lateral hypothalamus, project directly to reward centers like the VTA, linking homeostatic needs with hedonic feeding drive (92, 93). Recent research indicates that orexin neuron activity mediates mice’s choice between palatable food and voluntary exercise, prioritizing exercise, highlighting the critical role of this system in balancing different reward options (94).

Although traditionally viewed as a motor control center, the cerebellum is now implicated in reward processing (95–97), emotions (98, 99), and cognitive functions (100, 101). Through extensive connections with the cortex and basal ganglia, the cerebellum may modulate reward prediction and learning (102, 103) (Table 1).

**Table 1:** Core brain regions and their functional roles in food decision-making.

Brain region	Primary neurobiological function	Specific role in dietary behavior
Nucleus Accumbens (NAc)	Interface between cognition, emotion, and action.	Integrates multi-regional information to optimize behavioral choices and drive “wanting.”
Ventral Tegmental Area (VTA)	Hub for the mesolimbic dopamine system.	Encodes reward prediction errors and motivational salience to drive food-seeking behavior.
Orbitofrontal Cortex (OFC)/vmPFC	Evaluation and representation of reward value.	Encodes relative preferences and dynamically updates food value based on internal states like satiety.
Dorsolateral Prefrontal Cortex (dlPFC)	Executive function and top-down inhibitory control.	Suppresses impulsive responses to tempting, unhealthy foods to facilitate long-term health goals.
Anterior Cingulate Cortex (ACC)	Performance monitoring and cost–benefit analysis.	Allocates cognitive resources and regulates the effort required to obtain specific food rewards.
Amygdala	Processing of emotional and motivational significance.	Facilitates the learning and memory of reward-associated cues and influences attentional bias.
Hypothalamus	Central regulator of energy homeostasis.	Integrates peripheral signals (leptin/insulin) and links homeostatic needs with hedonic drives via orexin neurons.
Cerebellum	Modulation of reward prediction and learning.	Contributes to cognitive and affective processing related to food rewards through extensive cortical connections.

### 2.2 Key neurotransmitters and hormones

As noted above, dopamine is more strongly implicated in the motivational component of reward, or “wanting,” than in hedonic pleasure itself (104–106). Its major function is to encode incentive salience and reward prediction, thereby energizing food seeking and cue responsiveness. Experimental findings further suggest that even when dopamine synthesis is impaired, basic hedonic preference for sweet taste may remain relatively preserved (107), indicating that dopaminergic dysfunction is more likely to reduce motivation to obtain food than the capacity to experience pleasure from it (105, 106).

Ghrelin, a primary “hunger hormone” secreted by the stomach, acts on the hypothalamus to stimulate appetite and directly modulates dopamine neurons in the VTA, thereby enhancing the rewarding value of food (108–110). Recent research has unveiled the complexity of this system. For instance, the endogenous ghrelin receptor antagonist LEAP2 has been found to reduce the intake of highly palatable foods and attenuate food-related dopamine release in the nucleus accumbens (111). Moreover, des-acyl ghrelin (DAG) has been shown to diminish alcohol reward, suggesting that different members of the ghrelin family may play distinct regulatory roles in the reward system (112).

**Figure 2:**
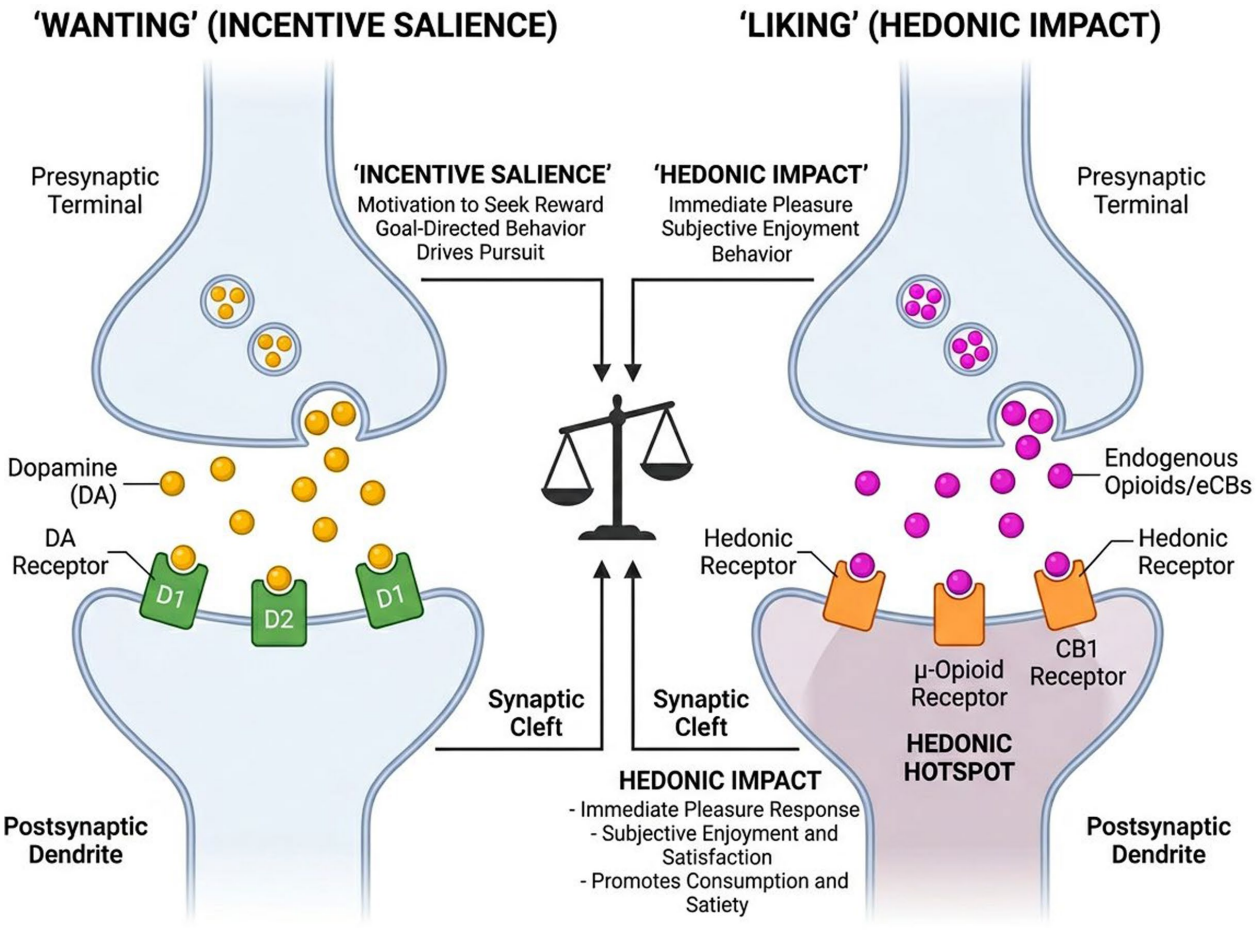
Neurochemical dissociation of “wanting” and “liking” systems. Schematic representation of the distinct neural pathways mediating food reward. “Wanting” is primarily driven by the dopaminergic system (yellow), encoding incentive salience. In contrast, “liking” (hedonic pleasure) is mediated by opioid and endocannabinoid signaling (magenta) within specific hedonic hotspots in the nucleus accumbens.

The endocannabinoid system, crucial in regulating hedonic eating, is influenced by the consumption of high-fat, high-sugar foods (113–115), which stimulate the synthesis of endogenous cannabinoids like anandamide and 2-AG in the brain. These substances, acting on CB1 receptors, enhance food palatability and dopamine release, establishing a “tasty loop” that may lead to overeating (116–120) (Figure 2).

### 2.3 Core cognitive processes

*Valuation and decision-making*: When faced with multiple food choices, the brain must assign a subjective value to each option and make decisions based on this assessment (121–123). This process is primarily governed by the OFC and vmPFC, which integrate the sensory characteristics of food (taste, texture), past experiences, current physiological state, and potential outcomes (124–126).

*Inhibitory control*: In the context of executive function, inhibitory control refers to the ability to suppress inappropriate or impulsive behaviors (127). Within the realm of dietary behavior, inhibitory control is crucial for resisting the temptation of high-calorie foods and adhering to long-term dietary goals. The PFC, particularly the dlPFC and ACC, serves as the neural basis for inhibitory control (72, 128). Variability in inhibitory control capacity among individuals may account for why some people are more susceptible to food cues.

*Learning and memory*: Food preferences are largely formed through associative learning (129). Pavlovian conditioning associates neutral environmental cues (such as restaurant signs, food packaging) with the rewarding value of food, independently triggering “cravings” and eating behavior (130–132). The hippocampus and amygdala play central roles in this learning and memory consolidation process (133, 134).

*Attention*: The attentional bias of the brain towards food-related cues is another important cognitive component of reward processing. Cues of highly palatable foods can automatically capture attention, making it harder for individuals to disengage attention from these temptations (135). This attentional bias is considered a key factor in driving food cravings and impulsive eating behaviors (136, 137). Eye-tracking studies have also revealed how cognitive processes guide the selection of visual information, which is particularly crucial in natural behavior (138).

## 3 Multifaceted influencing factors of food preferences and reward processing

**Figure 3:**
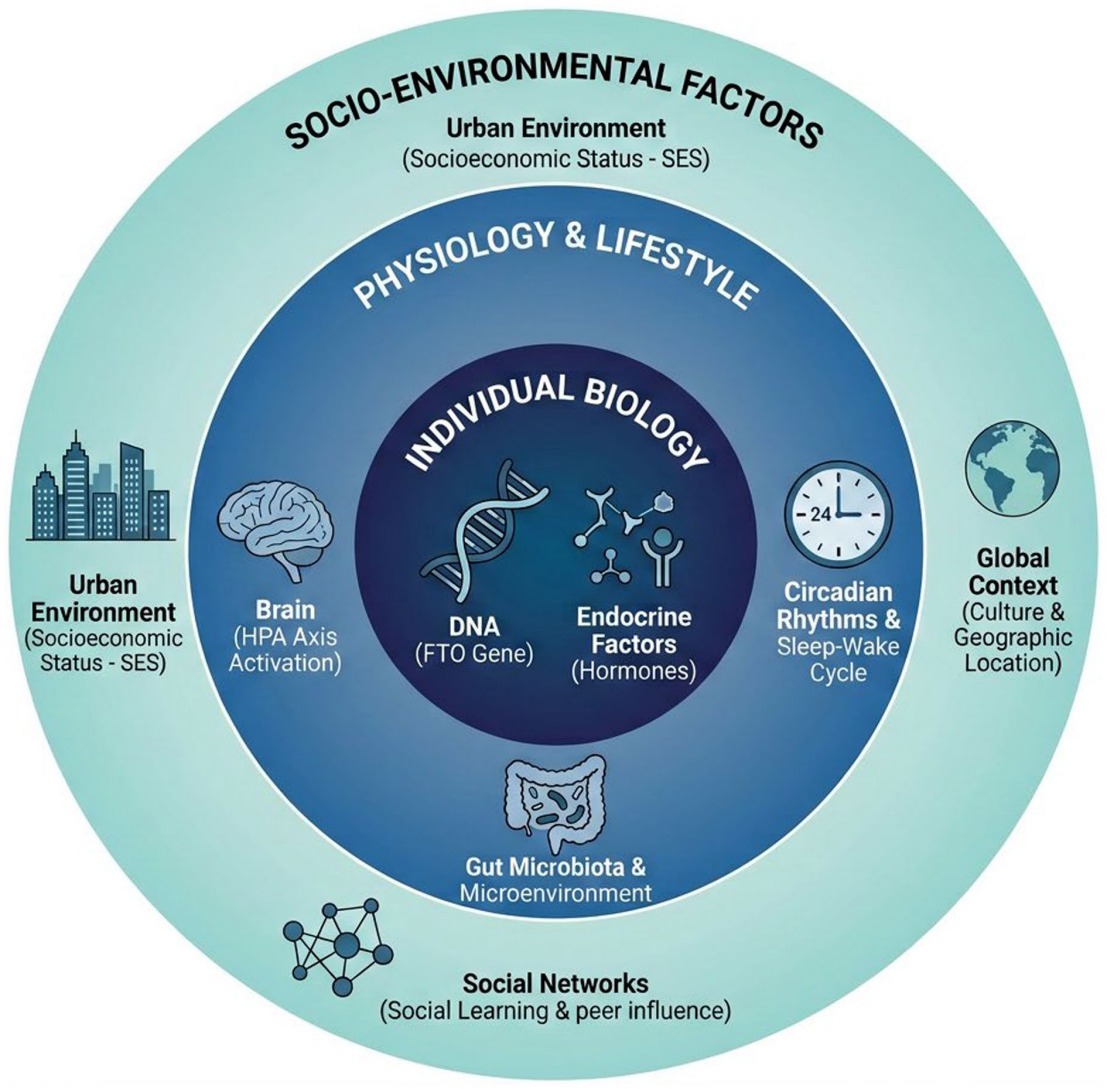
Hierarchical and interactive framework of determinants of dietary behavior. The figure summarizes how food choice is shaped by influences operating at multiple levels, from genetic and physiological factors to family, socioeconomic, and cultural environments. Importantly, these levels should not be interpreted as separate explanatory layers. Rather, they interact dynamically: contextual factors shape exposure, reinforcement, and stress burden, while biological and cognitive characteristics influence sensitivity to food cues, valuation processes, and inhibitory control. The framework therefore highlights how eating behavior emerges from cross-level interactions rather than from any single determinant alone.

Individual food choices are made within a complex biological, psychological, and social context. Various factors interact to shape the brain’s response to food rewards (Figure 3).

### 3.1 Individual factors

*Genetic Factors*: Genetics play a crucial role in determining food preferences and susceptibility to obesity (139–142). For instance, variations in the FTO gene linked to obesity risk (143, 144) have been associated with dysregulation of ghrelin levels and reduced responses in the brain’s reward regions to food cues, potentially leading individuals to require greater food intake to achieve the same level of satisfaction (145, 146).

*Physiological States and Circadian Rhythms*: Hunger, satiety, and related physiological states directly alter the reward value of food (147, 148). The circadian system also regulates metabolic and appetite-related hormones (149, 150), thereby shaping food preference (151) and food intake at different times of day (152).

*Age and Development*: During the lifespan, food preferences and reward processing undergo continuous changes (153, 154). Adolescence represents a period of significant neural remodeling, particularly in the PFC, leading to alterations in emotional regulation and cognitive control, potentially increasing impulsive choices for highly rewarding foods (155, 156). Conversely, in older adults, factors such as taste perception (157) and chewing functionality (158) may become more critical determinants of food preferences, although their direct association with cognitive function remains unclear (159).

*Stress*: Acute and chronic stress significantly impact dietary behavior by altering the sensitivity of the reward system and weakening the cognitive control function of the PFC, leading individuals to prefer high-fat, high-sugar “comfort foods” (10, 160, 161). Interestingly, the consumption of these foods can, in turn, buffer the stress response of the hypothalamic–pituitary–adrenal (HPA) axis, creating a potential vicious cycle (36). Social stress experienced during adolescence may even have lasting negative effects on cognitive functions in adulthood, possibly linked to impaired development of the mesocortical dopamine system (162–164).

*Health Status (Obesity and Psychiatric Disorders)*: Obesity induces alterations in both the structure and function of the brain (165). Individuals with obesity typically exhibit heightened responses in reward-related brain regions to food cues, alongside decreased activity in cognitive control regions (166). Moreover, obesity-related systemic low-grade inflammation may lead to neuroinflammation, particularly in critical regions like the hypothalamus, further impairing cognitive function and energy homeostasis regulation (167). Beyond endocrine signaling, the gut microbiota serves as a critical transducer of dietary signals into neurobiological responses (168). Specifically, microbial metabolites such as short-chain fatty acids (SCFAs) exert anorexigenic effects by modulating hypothalamic pro-opiomelanocortin (POMC) neurons via vagal afferent signaling or direct systemic circulation. Furthermore, dysbiosis-induced intestinal permeability may exacerbate neuroinflammation in reward-related hubs, thereby altering the sensitivity of the homeostatic-hedonic crosstalk that governs long-term energy balance. Psychiatric disorders such as schizophrenia are often accompanied by abnormalities in reward processing and motivational deficits, potentially influencing patients’ eating behaviors and weight (169).

Importantly, these individual-level determinants do not operate in isolation. Their effects are continuously amplified, buffered, or reshaped by social and environmental context. In other words, food-related reward processing emerges from a dynamic interaction between internal susceptibility and external exposure: biological predispositions influence how individuals respond to food cues, while socioeconomic conditions, social learning, and cultural norms affect which cues are encountered, how often they are reinforced, and whether cognitive control can be effectively deployed. This interactional perspective helps link individual neurobiology with broader real-world eating environments.

### 3.2 Social and environmental factors

Social and environmental influences do not act merely as background conditions for eating behavior; they shape food-related decisions through identifiable neurocognitive pathways. By altering chronic stress exposure, learned cue associations, attentional salience, and the demands placed on self-regulation, contextual environments can modify how reward-related regions, valuation systems, and executive control networks respond to food. The following factors should therefore be understood as interacting with, rather than operating separately from, the neurobiological mechanisms outlined above.

*Socioeconomic status (SES)*: Lower SES is often associated with poorer diet quality and with developmental conditions that can shape long-term health and cognitive outcomes (170–172). Research indicates that the SES gradient can forecast individual differences in children’s neurocognitive abilities, such as language and executive functions (173), which may impact their understanding of nutritional information, planning of healthy diets, and ability to resist unhealthy food temptations.

*Social Learning and Emotional Contagion*: Humans, as social animals, acquire their food preferences largely through observation and imitation of others, especially family members and peers (174). Studies on animals indicate that rodents can learn preferences for new foods from the odors of their peers through the “social transmission of food preferences” paradigm (42). This social learning mechanism may be more complex in humans, involving cultural and emotional factors.

*Culture and Gender*: In the context of diverse cultural backgrounds, significant disparities exist in the symbolic meanings of food, culinary practices, and dietary habits, all of which profoundly shape individual food preferences (175, 176). Moreover, within the same culture, notable gender differences are present. A large-scale study conducted on the Italian population revealed that men tend to prefer red meat and processed meats, while women show a greater inclination towards healthier food choices such as vegetables, whole grains, and dark chocolate (177). These variations may stem from a combination of physiological needs, societal expectations, and gender roles.

*Significant Societal Events*: Large-scale societal disruptions, such as the COVID-19 pandemic, can also reshape food preferences and food-related decision-making. A longitudinal study of Chilean students found greater sensitivity to food costs during the pandemic, together with a stronger preference for foods perceived as healthier and more environmentally sustainable, possibly reflecting heightened awareness of health risks (178).

## 4 Intervention strategies

Based on an understanding of food preferences and reward processing mechanisms, researchers and clinicians are developing various intervention strategies aimed at assisting individuals in establishing healthier eating habits.

### 4.1 Pharmacological intervention

In recent years, significant progress has been made in developing drugs targeting the gut-brain axis. Glucagon-like peptide-1 (GLP-1) analogs, such as semaglutide and liraglutide, initially used for type 2 diabetes treatment, have emerged as effective weight-loss medications. These drugs not only suppress appetite by acting on the hypothalamus but may also directly impact the brain’s reward pathways. Clinical studies indicate that GLP-1 analog therapy can reduce energy intake, decrease preference for high-fat foods, and enhance feelings of dietary control (179). However, further research is needed to understand the long-term effects of these drugs in weight maintenance and their specific neural mechanisms influencing eating behavior (180). Other drugs targeting neurotransmitter systems, such as the opioid system, are also under investigation, although their use is often limited by side effects (181).

### 4.2 Behavioral and cognitive interventions

Physical exercise not only increases energy expenditure but also positively impacts brain function. Research indicates that acute high-intensity interval exercise (HIIE) can enhance inhibitory control and working memory in young women, with the improvement in inhibitory control mediating the exercise’s positive effects on food choices (182). Long-term exercise can also improve executive function and reward processing in patients with depression through mechanisms such as increasing brain-derived neurotrophic factor (BDNF) (72). Furthermore, the hypothalamic orexin system, as mentioned earlier, plays a role in balancing exercise and food rewards, providing a new neurobiological perspective on how exercise may help individuals resist food temptations (94).

### 4.3 Surgical intervention

**Figure 4:**
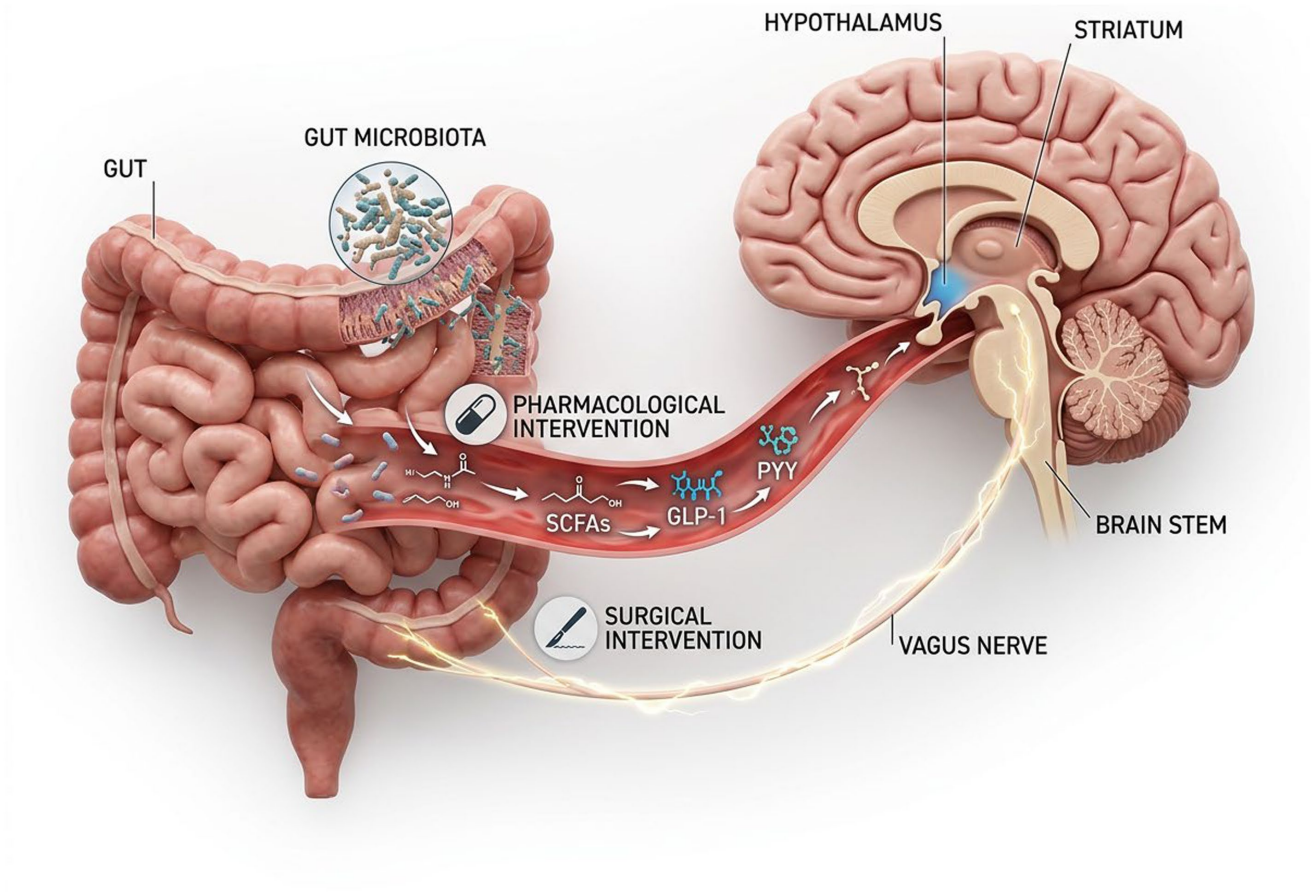
The gut-brain-microbiota axis as a therapeutic target. The figure depicts bidirectional communication between the gastrointestinal system and the brain through neural, endocrine, immune, and metabolic pathways. Microbial metabolites and gut-derived hormones can influence hypothalamic homeostatic regulation as well as reward-related responses to food cues, partly through vagal and systemic signaling. The diagram also indicates where major interventions—including GLP-1-based pharmacotherapy and bariatric surgery—may act within this axis, thereby linking peripheral physiological changes to central alterations in appetite, valuation, and food-seeking behavior.

For severely obese individuals, bariatric surgeries such as Roux-en-Y gastric bypass and vertical sleeve gastrectomy are the most effective long-term weight loss methods. These surgeries work not only by restricting stomach capacity and reducing nutrient absorption but also by altering the secretion of gut hormones (e.g., GLP-1, PYY), profoundly impacting gut-brain axis signaling. A systematic review of functional magnetic resonance imaging (fMRI) studies indicates that following bariatric surgery, regions in the brain associated with reward processing (e.g., striatum, insula) show reduced reactivity to high-calorie food cues, while activity in regions linked to cognitive control (e.g., dlPFC) may increase (166). These neural changes are closely associated with postoperative decreases in appetite, shifts in food preferences towards healthier options, and significant weight loss (Figure 4).

### 4.4 Other intervention strategies

*Sensory Intervention*: The sensory characteristics of food, such as taste, aroma, and texture, play a crucial role in determining its hedonic value. Enhancing the sensory experience of food, for instance, by boosting umami taste in the diet of elderly individuals or those with swallowing difficulties, may help increase their acceptance and consumption of nutritious foods, thereby improving their health status. Furthermore, some studies have also highlighted the benefits of nutrients like docosahexaenoic acid (DHA) on brain function and cognitive development, which could indirectly influence long-term dietary choices and health outcomes (Table 2).

**Table 2:** Comparative analysis of intervention strategies for food preference and reward dysregulation.

Intervention category	Representative modality	Core mechanism of action	Observed clinical/Behavioral outcomes
Pharmacological	GLP-1 Analogs (e.g., Semaglutide)	Modulates gut-brain axis signaling and impacts central reward pathways.	Reduced energy intake, decreased preference for high-fat foods, and enhanced dietary control.
Behavioral and Cognitive	Physical Exercise	Enhances inhibitory control, improves executive function, and increases BDNF levels.	Directly mediates healthier food choices by strengthening top-down cognitive regulation.
Surgical	Bariatric Surgery	Reshapes gut hormone secretion (GLP-1, PYY) and alters neurobiological reward sensitivity.	Reduced neural reactivity to high-calorie cues and increased activity in cognitive control regions.
Sensory and Nutritional	Umami Enhancement; DHA Supplementation	Optimizes the hedonic value of nutritious options and supports overall brain function.	Improved acceptance and consumption of nutritious foods, particularly in elderly or clinical populations.
Digital and Personalized	JITAIs via Digital Phenotyping	Utilizes machine learning and biosensors to identify idiosyncratic “vulnerability windows.”	Delivers real-time cognitive support precisely when inhibitory capacity is most compromised.

Taken together, these interventions can be understood as targeting different nodes within the reward-control system described earlier in the manuscript. Pharmacological approaches primarily act on homeostatic and neurochemical signaling pathways, especially those linking the gut, hypothalamus, and reward circuitry. Behavioral and cognitive interventions mainly aim to strengthen executive control, reduce cue-driven responding, and reshape learned associations. Bariatric surgery produces broader physiological and neural changes, altering gut-hormone signaling as well as food-cue reactivity in reward-related regions. Sensory and nutrition-based approaches may modify the hedonic and perceptual dimensions of food experience, thereby influencing valuation and acceptance. Framing interventions in this mechanistic way helps clarify why combined or personalized approaches may be more effective than single-component strategies.

## 5 Discussion and future perspectives

This review brings together evidence indicating that eating behavior is shaped by the interaction of homeostatic regulation, hedonic reward processing, and executive control. Rather than being driven by a single neural substrate, food choice appears to emerge from coordinated activity across multiple systems, including mesolimbic motivational circuits, cortical valuation networks, hypothalamic metabolic signaling, and prefrontal control mechanisms. A central implication of this framework is that maladaptive eating is best understood as a systems-level imbalance: excessive cue-driven “wanting,” heightened valuation of palatable foods, impaired inhibitory control, or altered homeostatic signaling may each contribute, and in many individuals these mechanisms likely co-occur.

At the same time, these neurobiological processes are embedded within broader developmental, psychological, and social environments. Genetic variation, age-related neurodevelopment, stress exposure, and metabolic status may alter sensitivity to reward and control demands, while socioeconomic conditions, cultural norms, and social learning shape the availability, meaning, and reinforcement of food cues in daily life. This cross-level view is important because it helps explain why similar food environments do not affect all individuals in the same way, and why effective intervention is unlikely to rely on a purely biological or purely behavioral model alone.

Several important issues remain unresolved. First, although the distinction between “wanting” and “liking” is conceptually useful, the empirical literature is still uneven across methods, populations, and behavioral phenotypes. In some domains, especially obesity and stress-related eating, findings are not always fully consistent, partly because studies differ in task design, outcome measures, and the degree to which homeostatic, affective, and contextual variables are controlled. Second, the neural mechanisms through which social and environmental influences alter food reward remain incompletely understood. Social modeling, emotional contagion, and socioeconomic adversity likely affect valuation and control processes, but the relevant pathways have not yet been mapped with sufficient precision. Third, more integrative research designs are needed. Future work should more directly combine neuroimaging, ecological assessment, longitudinal follow-up, and computational or mechanistic modeling in order to capture how food-related decisions unfold across time and context (183). Finally, intervention research would benefit from stronger personalization frameworks. Different individuals may show different combinations of reward hyper-reactivity, impaired control, stress sensitivity, metabolic dysregulation, or social vulnerability, suggesting that stratified or multi-component approaches may be more effective than uniform interventions.

**Figure 5:**
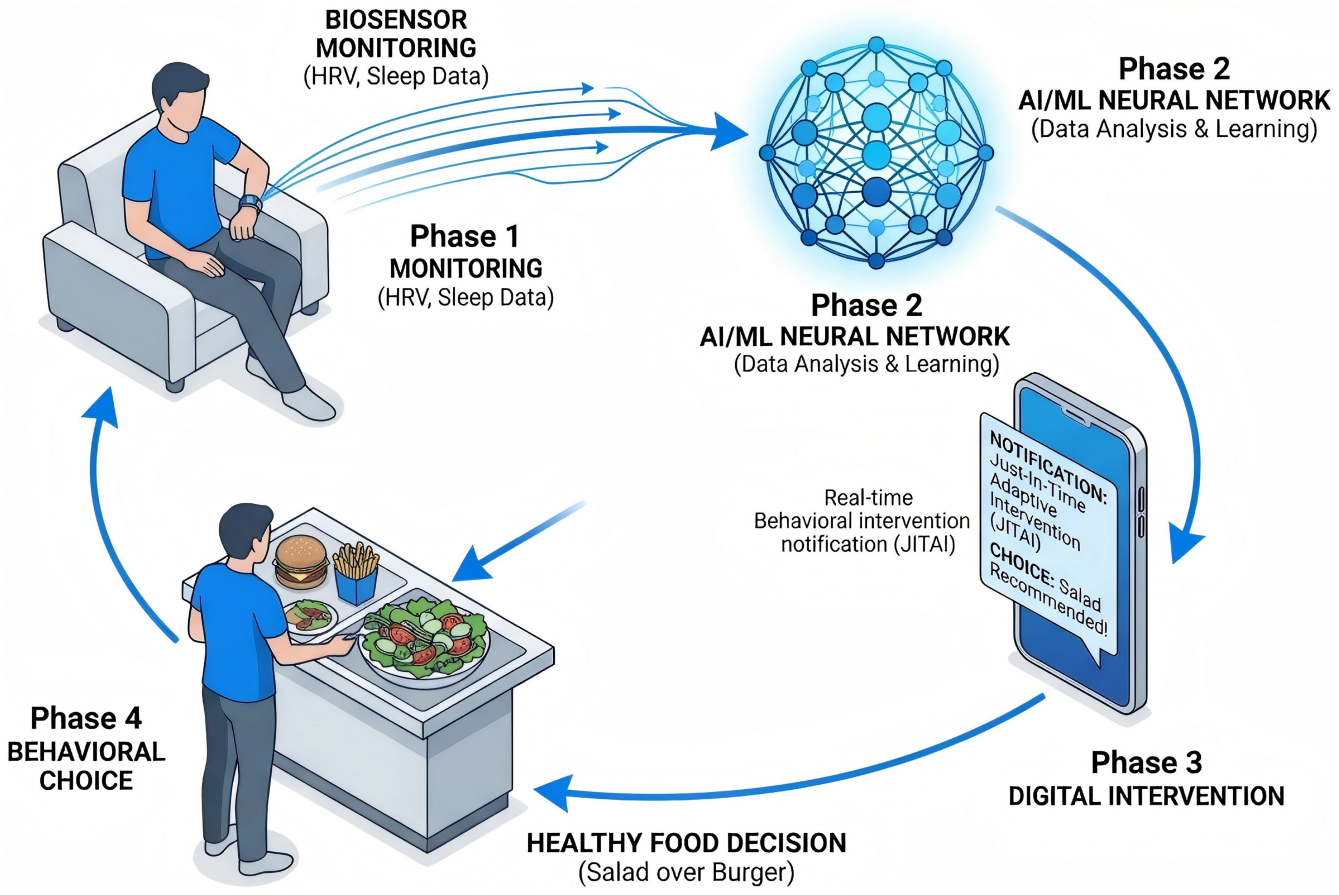
Personalized intervention framework based on digital phenotyping and just-in-time adaptive interventions (JITAIs). The figure presents a conceptual model in which wearable and mobile data streams are used to identify temporally specific windows of vulnerability for maladaptive eating, such as periods of elevated stress, poor sleep, or reduced self-regulatory capacity. In translational terms, the framework links real-world monitoring to timely support delivery. At present, this framework should be regarded as a promising translational direction rather than an established clinical solution, and further evidence is needed to determine its long-term effectiveness, generalizability, and scalability in nutritional and eating-behavior contexts.

Emerging digital health approaches may help operationalize such personalization in real-world settings. Digital phenotyping based on wearable sensors, ecological momentary assessment, and mobile behavior tracking offers a practical way to detect fluctuations in stress, sleep, affect, and eating-related vulnerability. In principle, these data streams could support Just-In-Time Adaptive Interventions (JITAIs), in which support is delivered when self-regulatory capacity is low or cue reactivity is elevated. However, the current evidence base in nutritional neuroscience remains preliminary. Although feasibility is increasingly plausible, stronger prospective and intervention studies are still needed to determine whether these approaches produce durable improvements in eating behavior, adherence, and metabolic outcomes across diverse populations (Figure 5).

## 6 Conclusion

Food preference and reward processing are best understood as the products of interacting neural, physiological, cognitive, and social processes rather than as isolated functions of a single brain region or pathway. The literature reviewed here suggests that maladaptive eating may arise from multiple forms of imbalance within the broader reward-control system, including altered motivational salience, disrupted valuation, impaired inhibitory control, and dysregulated homeostatic signaling. This perspective helps explain both the heterogeneity of eating-related problems and the need for interventions that are mechanistically informed and context sensitive. Looking forward, the most important challenge is to move from descriptive models toward integrative and personalized frameworks that can better connect neurobiological insight with real-world prevention and treatment.
